# Pharmacist and patient perspectives on recruitment strategies for randomized controlled trials: a qualitative analysis

**DOI:** 10.1186/s12874-020-01140-6

**Published:** 2020-10-31

**Authors:** Jane M. Fletcher, Terry Saunders-Smith, Braden J. Manns, Ross Tsuyuki, Brenda R. Hemmelgarn, Marcello Tonelli, David J. T. Campbell

**Affiliations:** 1 Department of Ecosystem and Public Health, Faculty of Veterinary Medicine, University of Calgary, Calgary, AB T2N 1N4 Canada; 2 Department of Medicine, Cumming School of Medicine, University of Calgary, Calgary, AB T2N 1N4 Canada; 3 Department of Community Health Sciences, Cumming School of Medicine, University of Calgary, Calgary, AB T2N 1N4 Canada; 4 Department of Pharmacology, Faculty of Medicine and Dentistry, 2J2.00 WC Mackenzie Health Sciences Centre, University of Alberta, 8440 112 St. NW, Edmonton, AB T6G 2R7 Canada; 5 Department of Cardiac Sciences, Cumming School of Medicine, University of Calgary, Calgary, AB T2N 1N4 Canada

**Keywords:** Recruitment, Qualitative, Randomized controlled trials, Pharmacist, Participant focus group, Chronic disease, Medication, Education, Senior

## Abstract

**Background:**

Although recruitment is a major challenge for most randomized controlled trials, few report on the difficulties of recruitment, or how it might be enhanced. The objective of our study was to qualitatively explore the experiences of both patients and pharmacists related to recruitment for ACCESS, a large trial involving low-income seniors, given that two of our most successful recruitment strategies were direct patient recruitment materials and use of community pharmacists.

**Methods:**

Using qualitative descriptive methods, we collected data from pharmacists and study participants. Pharmacists were asked about their impressions of the study, as well as challenges they faced and methods they used to recruit potential participants. Focus groups with trial participants centered on the patient recruitment materials. Interviews and focus groups were recorded, transcribed and analyzed using thematic analysis.

**Results:**

Pharmacists noted that their first impressions of the study were positive as they described being enticed to help the study team by the potential benefit of copayment elimination for their patients and the low time commitment. Pharmacists noted they were more likely to recruit if they were well informed on the study, as they could answer their patients’ questions. Participants noted that their primary motivations for participating were the tangible benefits of free medications and the intrinsic value of participating in research.

**Conclusions:**

We noted that recruitment through pharmacies was an effective method as most patients have trusting relationships with their pharmacist. To optimize recruitment through pharmacies, study procedures should be straightforward, and pharmacists need to be equipped with good knowledge of the study. When promoting a study to potential participants, messaging should ensure the individuals are aware of the tangible benefits of participation while still presenting a full overview of the trial.

**Trial registration:**

Trial Registration Number: NCT02579655 – initially registered Oct 19, 2015.

## Background

Randomized controlled trials (RCTs) are considered the most robust study design in health research and can reduce much of the bias inherent in observational study designs [1]. Investigators often underestimate the time, costs, and difficulties involved with recruiting trial participants, as only 55% of RCTs reach their pre-specified recruitment targets [2]. Difficulties with recruitment can lead to delays, cost overruns [3], and potentially a resultant lack of statistical power [4]. Several review articles have highlighted the need for improved recruitment efficiency with suggestions for general techniques to enhance recruitment such as phone call reminders, ‘opt-out’ recruitment methods, or financial incentives, but these methods are not universally applicable [5–7].

Health care professionals outside of the immediate research team can be effective in recruiting patients due to pre-established relationships with patients who are potential study participants [8, 9]. Pharmacists have been suggested to be especially helpful in recruiting participants [10, 11], particularly for community-based trials, or those that focus on medications or adherence.

Another means of recruitment used in many RCTs is patient-facing self-referral materials. It remains uncertain what types of messages are most impactful for attracting patients to enroll in research. Some studies have suggested that incorporating participant feedback when creating materials might assist in recruitment, as participants may provide insights that were not considered by study staff [12]. Obtaining participant input early on in studies could potentially save both money and time by improving recruitment efficiency [13].

## Methods

### Study aims

Since pharmacists and patient-facing materials were among the most commonly used methods for recruiting for our community-based clinical trial, our objective was to use qualitative methods to better understand participants’ and pharmacists’ experiences with the recruitment process within the context of a large community-based RCT. Additionally, given the importance of patient input into recruitment activities, we sought feedback on recruitment materials from participants in order to inform subsequent recruitment efforts. While the use of these particular methods to recruit participants for a randomized trial is not novel in itself, conducting a qualitative descriptive study to explore perceptions of both patients and recruiting pharmacists on recruitment processes is seldom reported and has the potential to make a meaningful contribution to the literature and enable these two strategies to be used more effectively in future research.

### Study context

The **A**ssessing outcomes of enhanced **C**hronic disease **C**are through patient **E**ducation and a value-ba**S**ed formulary **S**tudy (ACCESS) is an ongoing 3-year randomized controlled trial evaluating the impact of two interventions on cardiovascular endpoints in Alberta, Canada [14] (ClinicalTrials.gov Identifier: NCT02579655). Eligible participants were low-income (<$50,000 CAD/year) adults 65 years of age or older, with at least one cardiovascular-related chronic health condition, and Alberta Blue Cross Seniors’ Benefit coverage, allowing premium-free access to most medications with a 30% copayment. ACCESS is a factorial (2 × 2) trial studying the following interventions: (1) a medication copayment elimination plan whereby participants received all high value cardioprotective medications without copayment, and (2) a self-management education program individually tailored to each participant [14]. Recruitment for the ACCESS Trial began in November 2016 and concluded in September 2018, with follow-up ongoing, and expected to be complete in mid-2021.

Participants enrolled in the ACCESS study by receiving initial information about the study in the community – through a number of sources, including pharmacists, physicians, outreach activities and media. Regardless of the means by which a participant heard about the study, they were directed to call a central study telephone line where they were then screened for eligibility, after which they completed written informed consent and baseline questionnaires before being randomized. We recently published a detailed retrospective examination of the various recruitment strategies utilized to enroll more than 4000 participants in the ACCESS trial [15]. We found that pharmacists recruited 38% of participants. However, this strategy was also relatively resource intensive, costing over CAD$150,000. The vast majority of ACCESS participants were enrolled either through pharmacies (where recruitment materials were present) or by encountering recruitment materials such as brochures and posters in other locations such as community centres, medical offices, churches, seniors’ centres, recreational centres, and shopping malls – accounting for another 43% of our participants [15]. Given the importance of these two recruitment methods for helping us achieve our target sample size, we decided to focus this qualitative study on exploring these two methods in greater detail.

### Study design

This study used qualitative descriptive methods [16], which aim to describe phenomena in the everyday terms of the participants [17]. Our objective was to explore the perceptions of the recruiting pharmacists and trial participants with regards to the reasons underlying successes and challenges with recruitment in ACCESS. Both the ACCESS trial and this qualitative exploratory substudy have been approved by the University of Calgary Conjoint Health Research Ethics Board.

### Sampling & Data Collection

We collected data using individual semi-structured interviews (with pharmacists) as well as focus groups (with study participants). The pharmacists were sampled purposively [18] from the 855 pharmacies that recruited for ACCESS. Enrollment was limited to pharmacists who recruited at least one participant, but was not limited by geographic location within the province. The strata used for purposive sampling included the variables reported in Table 1. Sampling continued until saturation [19], meaning no new themes or codes emerged from additional interviews. Interviews, ranging in duration from 20 to 50 min, were conducted by telephone in the spring of 2018 [20] by a research assistant (JMF) who was trained in qualitative interviewing. JMF also recorded field notes. Interviews were recorded and professionally transcribed verbatim. A question guide was used to ensure similar material was covered during each interview (Supplemental Material 1). The pharmacists were informed of the purpose of the study at the enrollment and consent stage.

**Table 1:** Characteristics of Pharmacists (*n* = 20)

**Recruiter Significance**^a^	Major: 11
	Minor: 9
**Location**^b^	Urban: 8
	Rural: 12
**Type of Pharmacy**	Chain: 10
	Independent: 10
**Gender**	Female: 9
	Male: 11
**Additional Prescribing Authority**^c^ (unknown for one pharmacist)	Yes: 10
	No: 9

^a^Major defined as recruiting 3 or more participants into the study

^b^Urban: Calgary, Edmonton, Red Deer, Lethbridge, or Medicine Hat

^c^With special training and licensure, some pharmacists are eligible to prescribe medications in Alberta [21]

In the spring of 2017, two in-person focus groups, each lasting 2 h, were held with participants who had enrolled in ACCESS during the preceding 6 months. The purpose of these groups was to inform the researchers about the recruitment materials and to refine techniques and messaging. The two groups were drawn from the two intervention arms in the ACCESS trial; individuals within each group were selected through purposive sampling based on age, gender, and income. As the purpose was to obtain feedback on the recruitment materials, individuals recruited by pharmacists and by friends/family members [15] were not included, as they may have been told about the study without having seen the printed materials. Only participants situated close to Calgary were invited to minimize travel requirements. Focus groups were facilitated by TSS and DJTC (Supplemental Material 2). Focus group proceedings were digitally recorded and transcribed verbatim. Field notes were recorded during focus groups and written documentation was retained by the study team. The participants were informed of the purpose of the study at the enrollment and consent stage.

### Data analysis

Transcripts from interviews and focus groups were imported into NVivo 12 software (QSR International, Doncaster, Australia) for management of the qualitative data. Thematic analysis techniques were used to code the data [22]. The initial coding template was based on the interview guide, and additional categories were added inductively through open coding, which was completed independently by JMF and TSS, and then reviewed with DJTC. Focused coding was completed by reviewing all the codes and collapsing them into groupings.

## Results

### Study sample

Initially, 27 pharmacists were approached for an interview, with 20 agreeing to participate, and 7 declining due to time commitments or not meeting the criteria of having recruited at least one participant. After 20 interviews were completed, saturation had been reached and we decided to discontinue pharmacist interviews. The pharmacists had a relatively even distribution across the criteria used for purposive sampling (Table 1). 38 participants were contacted for the focus groups. Of those, 17 agreed to participate, and 14 attended the focus groups (Table 2). We present our results based on the type of participant: first barriers and facilitators related to pharmacists, followed by patient-level feedback. Results are supported by quotes in Table 3.

**Table 2:** Characteristics of Focus Group Participants^a^

Demographics		Copayment Elimination (***n*** = 8)	Personalized Education (***n*** = 6)
**Gender**	Female	4	4
	Male	4	2
**Age**	65–70	2	2
	71–75	2	3
	> 75	4	1
**Income**	<$15,000-29,999	5	4
	$30,000-50,000	3	2
**Marital status**	Married/Common-law	3	2
	Single/Other	5	4
**Highest level of education**	Post-secondary diploma or higher	3	4
	Less than post-secondary diploma	5	2
**Number of people living in household**	1	5	2
	≥2	3	4
**Country of birth**	Canada	4	5
	Other	4	1
**Native language**	English	6	5
	Other	2	1
**Health literacy**^b^	Adequate	7	5
	Inadequate	1	1

^a^Eligibility criteria for the ACCESS study include: (a) age greater than 65 years with Alberta government-sponsored seniors drug insurance, (b) high cardiovascular risk based on a history of any one of: heart disease, stroke, chronic kidney disease, heart failure, or any two of: current smoking, diabetes, hypertension, or high cholesterol; and (c) household income <$50,000. Exclusion criteria include: (a) coverage by a secondary insurance plan (in addition to Blue Cross), resulting in patient-borne copayment of < 30%, or (b) inability to participate in self-management modules due to cognitive impairment or a lack of an English-speaking family member or close friend [14].^b^Using validated single item screening tool [23]

**Table 3:** Quotes from Qualitative Interviews and Focus Groups

Pharmacist-Related Feedback	
**Study Introduction**	
ACCESS Study staff	*“[ACCESS Study staff] called me and then you sent information, pamphlets and this kind of things and then we displayed on the counters.”* (Pharmacist 15)
Colleagues	*“A colleague [pharmacist] told me about [the ACCESS Study].”* (Pharmacist 14)
Corporate managers	*“Somebody from the ACCESS study had reached out to one of the district managers of Rexall and they sent an email out to every single store that we might be getting a package.”* (Pharmacist 17)
Patients/Participants	*“Some of our patients told us they are enrolled into [the ACCESS Study] and their medications are going to be basically 100% covered, that’s when I first heard about it.”* (Pharmacist 10)
**Barriers**	
Understanding of the study	*“I don’t know very much about [the education program] at all, not many patients have spoken to us about it”.* (Pharmacist 10) *“I didn’t know that was an intervention. I thought [the education] was available to all patients in the study*.” (Pharmacist 18)
Lack of patient follow-through	*“I do provide information, but unfortunately I never follow up with it, if they filled out a form and send it or not because I know from statistical data a lot of people who do see things they don’t follow-up on it”* (Pharmacist 17)
Staff engagement	*“if the [pharmacy staff member] just giving [the patient] flyers is not even engaged enough to know like the intricate details about the things you are telling me about”* (Pharmacist 7)
Lack of time	*“I feel so limited because of how tight my days are and I’m also an associate owner of the [pharmacy name], so like between being a pharmacist and then all the managerial admin stuff it’s almost impossible to do anything”* (Pharmacist 7)
Displaying of materials	*“Posters around here have short life. They get leaned on, bumped, torn, shop-worn. We don’t really have a good area for posters, so as I say, they are on the counter they get ripped, torn and dirty.”* (Pharmacist 20)
**Facilitators**	
Benefit to patients	*“I thought it was a great idea, especially since we have a high demographic of seniors out here, and often hear complaints about costs of medication*” (Pharmacist 1)
Scientific merit	*“I certainly appreciate the efforts of the people that are doing research. I’m not one myself, to be up for doing that, so I’m always happy to help … I think it’s really important to continue coming up with data as far as what is affecting patients, and what pharmacies can do to help”* (Pharmacist 13)
Ease of participation	*“I just thought it sounded like it would be really easy for me. A really easy way for me to engage in research … I feel so limited because of how tight my days are, but my initial thoughts when I heard about [ACCESS] was that sounds like a convenient way for me to [get involved]”* (Pharmacist 7)
**Methods**	
Blanket strategies	“*I have handed [brochures] like crazy to every single person who is about 65*” (Pharmacist 17) “*Any senior that comes in to fill that does meet the criteria, we would talk to them about the study*” (Pharmacist 19)
Targeted strategies	Those with financial barriers: *“Some patients have a lot of issues with finance part of things, so that’s how we tailor to check what patient needs something”* (Pharmacist 10) Those needing education: *“[W] e tried to promote [ACCESS], it might help you both ways, education plus additional coverage as well”* (Pharmacist 15) Those with chronic conditions: *“if [a] senior had chronic conditions we promoted that, ‘you can try this [study], it might help you both ways, education plus it might help you with additional coverage as well’.* (Pharmacist 15) Those with poor compliance: “*Maybe they are struggling with filling prescription, they are not that compliant … those are the patients we are targeting most of the time”* (Pharmacist 3)
Self-directed strategies	Radio advertisements: “*we ran radio ads here and tried to attract as many people as we could for you”* (Pharmacist 20) Print & social media advertisements: *“I had it in the newspaper and then we went online with it as well too, Facebook and our other two Facebook pages that we have”* (Pharmacist 20) Community events: *“I have presented at senior events and I have just spoken to people and handed out them the pamphlet*” (Pharmacist 4)
**Participant-Level Feedback**	
**Barriers**	
Understanding of the study	*“I really don’t like the computer. I’m not a fan. Talking to somebody [on the phone] you can ask questions. If you are on a website, you kind of have to bumble around and try to figure it out”* (Patient 2)
Language	*“Because we have so many in Calgary “I think language barrier, because sometimes patients are intimidated for having to fill out forms or speaking to someone on the phone”, they may understand some English but not all”* (Patient 8)
Culture	*“the big thing is, people do not understand, when you look at the cultural groups, they are going to look at it and say, ‘well free, why is it free?’. Preventative, they don’t understand what that means”* (Patient 1)
Skepticism	*“University of Calgary, they are very credible, but some people would say, ‘if this is a medical study then why isn’t Health Calgary involved?’”* (Patient 1)
Logistics of enrollment	*“The biggest barrier that we had was some of the patients saying that they sometimes can’t get a hold of you”.* (Pharmacist 10)
Not seeing benefit of enrollment	*“[Some patients] don’t need any financial help, so they are not interested”* (Pharmacist 9)
Forgetting to enrol	*“I don’t have any reservations, I completely forgot about the study”* (Patient 6)
**Facilitators**	
Benefits of the interventions	Tangible benefit: Copayment elimination: “*The free medication because it is a benefit to me because it adds up, sometimes it’s up to $100 a month*” (Patient 9) Self-management education: “*I was wondering if there was something that they could maybe come up with that would improve my lifestyle, my conditions*.” (Patient 3) Societal benefit: “*Well actually I did want to participate you know, to benefit society, it didn’t really hit me about the free medication part until much later*.” (Patient 6) Personal benefit: “*I’m a curious person, I want to find out things, I’m not through learning yet”* (Patient 3)
Social support	Pharmacists: *“Somebody is mentioning the cost of their medications, [I would] say, ‘well you know this is something they are trying to discover and maybe you would get some of your medication paid for.’ That definitely seemed to be a good selling point. When people heard that part of it they would think that the phone call was worth their time and effort*” (Pharmacist 13) Friends and family: *“My daughter saw [a study promotion] and was all excited, she phoned me and said, mom there’s this study that you should participate in. So, she gave me the phone number and I called.”* (Patient 2)
Straightforward explanation	*“When you talk to seniors you’ve got to be straight up front”* (Patient 2)
**Feedback**	
Recruitment materials	Font size: *“You should make [the font on the brochures] bigger because that’s usually done when you are trying to hide something when you have the small print”* (Patient 6) Language inclusions: *“[The recruitment materials should] say all languages but officially our languages are English and French and if they need a translation that they can call and be advised in their language”* (Patient 8) Highlighting important study features: *“Could you highlight this area [looking for participants who are over the age of 65 and have Blue Cross”]”* (Patient 4)

### Pharmacist-level: barriers, facilitators, and recruitment methods

#### Pharmacists’ barriers to recruitment

A prerequisite for pharmacy recruitment is for pharmacists to hear about and understand the purpose of the study. There were some challenges disseminating knowledge about the study to the participating pharmacists. Nearly all pharmacists had a solid understanding of the objectives of the study: “*It was something to do with if there was a difference between people having coverage for chronic medications versus the ones that don’t … [with regards to] compliance and overall medical medication control” (Rx12).* However, there were apparent gaps in knowledge regarding the group allocation and enrollment procedures. Most had sufficient knowledge regarding the copayment elimination intervention, but fewer understood the education program.

One of the most common barriers that pharmacists cited was lack of patient follow-through once referred on to the study. They reported discussing the study with patients but suspected that many patients would not follow through with enrollment after their initial discussion. Pharmacists also discussed logistical issues with recruitment, including having to engage all other pharmacy staff members in recruitment. Another concern was a lack of time: *“you have such limited time with the patient, what are the things you are going to bring up and talk about and sometimes [the study]‘s one of them and sometimes it isn’t” (Rx13).* Some pharmacists also identified that the way materials were displayed within pharmacies limited their utility and presented a barrier.

#### Facilitators and strategies for pharmacist engagement

Having adequate knowledge about the trial was a key step in helping pharmacists recruit. Pharmacists described becoming aware of the ACCESS trial from a number of different sources including: (1) ACCESS study staff, (2) colleagues, (3) corporate management, and (4) patients/study participants.

In order to engage in the recruitment process, not only do pharmacists need to know about the proposed research, they must also see value in it. Many pharmacists described that they were interested in recruiting because the study offered additional support for their patients who were facing challenges. The education intervention was an added benefit for some pharmacists, but the main driver of engagement was the potential for patients to receive free medications. Some pharmacists also specified that they thought the intervention was likely to improve adherence: *“Sometimes they cannot afford the medications, they say ‘no I don’t want this one’. Sometimes they do not take it every day, just on and off. When the patient has to pay nothing, zero, they will show more compliance to the treatment” (Rx15)*. In addition to these tangible benefits to their patients, several pharmacists also expressed interest in the scientific merit of the study. Numerous pharmacists also mentioned that a factor in their decision to participate was the ease of participation, and the fact that very little was required of them, with the study team completing the enrollment procedures.

#### Methods for recruiting patients

Pharmacists utilized several methods for recruiting patients, which we categorized into ‘blanket strategies’ and ‘targeted strategies’. Blanket recruitment included handing out brochures to all patients in their pharmacy, or putting up posters in waiting areas: *“you know I have a corner there for people to wait for their prescriptions and have coffee and it has your poster there” (Rx14).* Targeted recruitment included speaking directly to potentially eligible patients. Often pharmacists put brochures directly in the medication bags of patients they thought might be eligible: “*When we filled prescriptions of some of the patients that we thought would be good candidates, we tuck in, so when they picked it up they got that” (Rx1).* The factors pharmacists considered in their targeting included: financial issues, lack of education, baseline chronic conditions, and adherence issues. They cited that they looked for these factors because of trial eligibility criteria and also because they knew that these patients were those who were most likely to be interested in enrolling in the trial.

Most pharmacists at least employed a blanket approach by putting out posters or brochures. Whether a pharmacist elected to additionally use a targeted approach depended upon whether he/she had trusting professional relationships with patients. Additionally, targeted approaches were easier for pharmacists who regularly undertook medication reviews with their patients*.* When pharmacists wished to delegate recruitment to a technician or student, they used only general, blanket approaches to recruitment, minimizing the need for detailed knowledge of the patients. Many pharmacists described using a combination of both blanket and targeted recruitment to introduce the study to patients: *“[Patients] would take off the tab and if I see somebody doing that or taking a picture of the poster I would hand them the leaflet and say, ‘here, keep this too and this is what the study is all about’ and just give them a rundown.”* (Rx17).

In addition to these routine techniques that were suggested by the ACCESS team, several pharmacists developed novel self-directed strategies to recruit patients, unbeknownst to the study team. These methods included using radio advertisements, newspapers and social media, and community events.

### Participant-level: barriers, facilitators, & recruitment material feedback

We categorized responses regarding patient-level experiences into similar themes as above: (1) patient-related barriers to recruitment; (2) facilitators to enrolment for participants; and (3) specific suggestions about the patient-facing study recruitment materials.

#### Participant barriers to enrollment

Many of the barriers described by patients, or perceived by pharmacists, stemmed from potential participants not fully understanding details about the study after their initial interactions. For example, one participant thought that he needed to access a computer in order to enroll, which was not the case. Language-related barriers contributed to this lack of knowledge, as study materials were only presented in English. Similarly, a participant identified that some individuals belonging to cultural and linguistic minorities may not have understood recruitment materials. Some participants were somewhat skeptical, or suspicious, about research. A pharmacist identified that some patients were concerned about the implications of being in a ‘trial’: “*[Patients’] initial ideas [about] any trial is that you will be popping pills that they don’t know” (Rx17)*. Another issue identified by both participants and pharmacists was that the benefits offered by the study might only appeal to those in need of financial assistance. A participant stated the study *“would only appeal to those whose costs aren’t covered” (Pt14)* and this sentiment was echoed by pharmacists as well. Finally, the logistical arrangement for recruitment contributed to participants’ difficulty enrolling, as patients needed to take the initiative to call the study enrollment centre. One of the more commonly identified barriers was that patients would forget about the study before having a chance to call in, which was also a concern identified by the pharmacists: *“they might seem gung-ho here, but by the time they get home and set it on the table, life goes on and you sometimes wonder how many people actually call”* *(Rx13).*

#### Facilitators to participant enrollment

The initial step in getting patients enrolled for a clinical trial is for researchers to generate interest within potential participants. Not unlike pharmacists, participants reported two major reasons for being interested in ACCESS: the direct benefits of the interventions, and the intrinsic value of the research. Most participants commented on the benefit of the additional medication coverage but, prior to enrollment, few participants saw the education program as being a major factor.

In addition to the direct benefits of the interventions, several participants mentioned that they chose to participate because of the value of the research – because it could benefit society, or because they derived some other benefit from it. Societal benefit included participants having altruistic motivations in advancing research that could benefit their peers. Personal benefit focused on the knowledge participants could gain through the study. Some participants expressed the opinion that these ‘intrinsic benefits’ were unlikely to be the true motivations behind participation, but that the underlying reason was the potential for receiving additional medication coverage: *“I mean we try to be holier-than-thou but it’s the free medication that we are all here for, and I think that’s going to be a big grabber for a lot of people*.” (Pt*2).*

Once individuals became interested, they still faced numerous challenges and barriers to following through with enrollment, as described above. Pharmacists reported using several strategies to help patients overcome these barriers. Rather than simply giving out brochures, pharmacists felt they were more likely to be successful by speaking with patients directly: “*if you just give it to them and don’t explain it, they are most likely not to phone the number that’s on there.” (Rx5).* Often the solution was to encourage the patient to call in, referencing the potential benefits of the medication coverage.

A number of other factors helped individuals overcome barriers to recruitment. Several participants recounted receiving encouragement from family members to become involved in the study*.* Others indicated that the involvement of a trusted institution engendered a sense of legitimacy: *“The University logo gives it credibility*.” *(Pt6).* This sense of trust was also seen with pharmacists: *“You see that it’s through the [University] so you know it’s a reputable study.” (Rx11)*. Furthermore, the straightforward and clear manner in which the study was explained to participants made them more likely to participate: “*[The article] was telling the facts, why you did the research and [how study staff] were going to go about it and it seemed to have no hidden agenda*” *(Pt13).*

#### Recruitment material suggestions

One purpose of the focus groups was to improve the appeal of the recruitment materials, therefore, participants were asked for direct feedback about the posters and brochures. While the overall impression was generally positive, there were concerns about various aspects of the materials. Some felt the messaging was not fully transparent about the fact that receiving medication coverage was not guaranteed. It was also noted that some of the language on the materials could be better explained, such as ‘senior’ (“*there are seniors 55 years old and it’s not going to cover them*” *(Pt3)*) and ‘chronic disease’. The small size of the font was also mentioned as a concern for this population*.*

Participants were asked to evaluate various strategies and messages. They preferred messaging promoting the possibility of a benefit to them (e.g. “*New study: providing free medication coverage & personalized health education”*) over messaging focused on empowering them to feel like they are contributing to something beyond themselves (e.g. “*Seniors can help improve health care costs”*). Participants preferred messaging about medication copayment coverage over messaging around the education program. Participants also preferred messaging that portrayed their individual importance to the research (e.g. “*Seniors needed*”) above more generic calls (e.g. “*Get involved*”).

## Discussion

Our findings support the importance of collaborative relationships between investigators and recruiting pharmacists. When engaging pharmacists to help with study recruitment, we learned that it is important to educate them on all facets of the study. In community-based trials like ACCESS, pharmacists helped recruit on a voluntary basis, and were not formal members of the study team, therefore it was not reasonable to expect them to know the study protocol as if they were team members. However, having a more in-depth knowledge of the study seemed to be beneficial to promoting recruitment. Both targeted and general recruitment strategies employed by pharmacists were thought to be effective. Pharmacists can help patients overcome hesitation to enroll in trials through direct conversations and encouragement. Patient-facing recruitment materials focusing on the potential tangible benefits were most impactful (as opposed to materials focusing on the social value of research). Study messaging needs to be clear and should not be perceived as misleading. Participants face a number of barriers to enrollment in research studies, and researchers can leverage clear communication techniques and the credibility of their institutions to engender trust with potential participants.

The use of pharmacists was an invaluable method of recruitment for the ACCESS trial [15], as has been documented in other trials [24]. Utilizing pharmacists allowed us to leverage pre-existing relationships they had with patients. Pharmacists who believed that involvement in the study would be beneficial to their patients were more motivated and effective recruiters. Because of their pre-existing trusting relationships with their patients [25–28], pharmacists were able to act as mediators in providing prospective participants with key information that enabled their recruitment. Most pharmacists were busy, yet many took the time to present the study to their patients in a positive manner due to the potential benefits for patients. When the benefit to patients is in question, others have found that recruitment is challenging and may potentially be compromised by the predetermined opinions of the providers tasked with recruitment [29]. Working with recruitment partners to address concerns and facilitate full support early on can also aid in recruitment, as suggested in our findings and in other studies [30]. The following considerations may enhance pharmacist-led recruitment: (1) The investigators must ensure that pharmacists are provided with the tools and opportunities to gain reasonable knowledge of the study aims and procedures; (2) Participation in recruitment should entail minimal workflow disruption; (3) There should be a potential benefit, both to patients and pharmacists; and (4) Closed-loop communication should exist between the study team and recruiting pharmacists regarding the enrollment of their patients. The utilization of numerous methods of introducing pharmacists to the study was beneficial, as several pharmacists described hearing about the study through multiple channels (e.g. study staff, colleagues, and patients). Pharmacists were a natural fit to assist with recruitment for the ACCESS trial, however, other health care professionals were also involved in recruitment to a lesser degree [15], and many of the principles outlined above would apply to these providers as well.

While involving pharmacists or other providers may help with enrollment, the importance of employing multiple strategies, such as direct advertising to the public, should not be overlooked. Studies have shown that most potential participants who encounter recruitment materials never actually enroll [31], although the reason why is rarely apparent [32]. Our study explored patients’ reasons for enrolling, and we suggest that investigators use clear communication strategies in recruitment materials, emphasize potential benefits to participants, and leverage the credibility of their institution’s reputation. Using a combination of these methods was found to be effective for the ACCESS trial. In particular, it can be useful for researchers to know what benefits would be of greatest interest to participants. In the ACCESS trial, the potential for enhanced medication coverage was considered to be a greater benefit than the educational program, and we found that recruitment materials focusing on the possibility of receiving this coverage were better received by potential participants. Several participants raised issues with recruitment materials because of language or cultural issues. Where possible, investigators should try to make recruitment messaging as accessible as possible for the target population. In the ACCESS trial, however, we decided that in order to benefit from the patient education intervention an individual had to be conversant in English anyway, so language tailoring of recruitment materials was not undertaken in our case.

Other clinical trials have examined the effects of their recruitment methods. Similar to our conclusions, one study recommended the use of plain, objective language, keeping personal biases out of discussions, and maintaining clear communication [33]. Another study found that travel inconveniences were a major reason for participants not wanting to become involved [34] – this was not an issue in the ACCESS trial as enrollment and follow-up was done remotely. Another study which explored recruitment qualitatively discussed the importance of obtaining patient feedback to improve recruitment early on in the trial, supporting our conclusions on the utility of this qualitative approach [30]. Others have noted that the main reason for participation in a clinical trial is self-interest – including financial incentives [35] with minimal evidence of altruistic motivation [36]. While one participant noted that it was likely that most participants were motivated by self-interest rather than altruism, many participants did cite altruistic motivations. The altruistic motivation is supported by previously published literature in which both the benefit to science and general altruism have been raised as reasons for study participation [37].

This study has some limitations. First, the main focus was on recruiting through pharmacists, specifically. However, the themes that emerged are likely true for engaging other health professionals who have existing relationships with potential participants. Second, it is possible that the established relationships between the pharmacists and the study staff might have influenced responses to the interview questions – however, it’s important to note that there were hundreds of pharmacies recruiting for ACCESS, so study staff had relatively infrequent contact with individual pharmacists. Third, the generalizability of our findings is limited, as the ACCESS study included only low-income seniors from Alberta who were being offered a chance to receive a direct financial benefit. Due to the fact that this study was conducted in Canada, where the healthcare system is unique in that universal coverage of pharmaceuticals is not guaranteed and those who have coverage often face copayments for medications [38] there may be limited applicability of the ACCESS intervention to systems outside of Canada. Whether these findings would apply to other trials in different contexts is unknown, but there are likely to be transferrable elements. Fourth, the relatively small sample size of the focus groups may have hampered our ability to achieve complete saturation. Finally, as the intention of the focus groups was to gain feedback on recruitment materials, participants who were recruited using other methods were excluded. This precluded us from obtaining patients’ detailed feedback on alternate recruitment methods (e.g. radio). Despite these limitations, this study adds to the previously published literature through the use of detailed qualitative methods, which have seldomly been used to investigate recruitment success and challenges [30]. We were able to capture richness and detail that is not possible in the quantitative studies or meta-analyses which have been previously published on this topic.

## Conclusions

Our findings will be of interest to investigators seeking to recruit a large number of older adults for pragmatic clinical trials. Future research should address recruitment processes across diverse demographic categories and study designs. In addition, investigators should consider including details regarding recruitment within publications, as few currently report on recruitment struggles. Considering that recruitment is a concern for all RCTs, proactively addressing these issues has the potential to decrease costs, increase enrollment, and reduce time taken to meet recruitment goals.

## Supplementary information

**Additional file 1.** **Interview Guide** - **A**ssessing outcomes of enhanced **C**hronic disease **C**are through patient **E**ducation and a value-ba**S**ed formulary **S**tudy (ACCESS).

**Additional file 2.** **Agenda** - ACCESS Study-Recruitment material Focus Group.

## Data Availability

The dataset used and/or analyzed during the current study may be made available from the corresponding author on reasonable request.

## Abbreviations

ACCESS: Assessing outcomes of enhanced Chronic disease Care through patient Education and a value-baSed formulary Study
RCT: Randomized Controlled Trial

## References

[1] Odgaard-Jensen J, Vist GE, Timmer A, Kunz R, Akl EA, Schünemann H, et al. Randomisation to protect against selection bias in healthcare trials. Cochrane Database Syst Rev. 2011;(4):MR000012 [cited 2019 Jan 6]. Available from: http://www.ncbi.nlm.nih.gov/pubmed/21491415. 10.1002/14651858.MR000012.pub3 PMC7150228 21491415

[2] Sully BGO, Julious SA, Nicholl J (2013). A reinvestigation of recruitment to randomised, controlled, multicenter trials: A review of trials funded by two UK funding agencies. Trials.

[3] McDonald AM, Knight RC, Campbell MK, Entwistle VA, Grant AM, Cook JA (2006). What influences recruitment to randomised controlled trials? A review of trials funded by two UK funding agencies. Trials.

[4] Altman DG (1980). Statistics and ethics in medical research: III How large a sample?. BMJ.

[5] Treweek S, Lockhart P, Pitkethly M, Cook JA, Kjeldstrøm M, Johansen M (2013). Methods to improve recruitment to randomised controlled trials: Cochrane systematic review and meta-analysis. BMJ Open.

[6] Caldwell PHY, Hamilton S, Tan A, Craig JC (2010). Strategies for Increasing Recruitment to Randomised Controlled Trials: Systematic Review. PLoS Med.

[7] Toledano MB, Smith RB, Brook JP, Douglass M, Elliott P (2015). How to Establish and Follow up a Large Prospective Cohort Study in the 21st Century - Lessons from UK COSMOS. PLoS One.

[8] Petosa RL, Smith L (2018). Effective Recruitment of Schools for Randomized Clinical Trials: Role of School Nurses. J Sch Nurs.

[9] Krusche A, Rudolf von Rohr I, Muse K, Duggan D, Crane C (2014). Williams JMG. An evaluation of the effectiveness of recruitment methods: the staying well after depression randomized controlled trial. Clin Trials.

[10] Tsuyuki RT, Al Hamarneh YN, Jones CA, Hemmelgarn BR. The Effectiveness of Pharmacist Interventions on Cardiovascular Risk: The Multicenter Randomized Controlled RxEACH Trial. J Am Coll Cardiol. 2016;67(24):2846–2854 [cited 2018 Sep 17]. Available from: https://www.sciencedirect.com/science/article/pii/S073510971632407X?via%3Dihub. 10.1016/j.jacc.2016.03.528 27058907

[11] Peytremann-Bridevaux I, Bordet J, Santschi V, Collet TH, Eggli M, Burnand B (2013). Community-based pharmacies: An opportunity to recruit patients?. Int J Public Health.

[12] DasMahapatra P, Raja P, Gilbert J, Wicks P (2017). Clinical trials from the patient perspective: survey in an online patient community. BMC Health Serv Res.

[13] Kaae S, Sporrong SK (2015). Patients’ reasons for accepting a free community pharmacy asthma service. Int J Clin Pharm.

[14] Campbell DJT, Tonelli M, Hemmelgarn B, Mitchell C, Tsuyuki R, Ivers N, et al. Assessing outcomes of enhanced chronic disease care through patient education and a value-based formulary study (ACCESS)—study protocol for a 2×2 factorial randomized trial. Implement Sci. 2015;11(1):131 [cited 2019 Jan 6]. Available from: http://implementationscience.biomedcentral.com/articles/10.1186/s13012-016-0491-6. 10.1186/s13012-016-0491-6 PMC5037634 27671037

[15] Kakumanu S, Manns BJ, Tran S, Saunders-Smith T, Hemmelgarn BR, Tonelli M (2019). Cost analysis and efficacy of recruitment strategies used in a large pragmatic community-based clinical trial targeting low-income seniors: A comparative descriptive analysis. Trials.

[16] Neergaard MA, Olesen F, Andersen RS, Sondergaard J (2009). Qualitative description – the poor cousin of health research?. BMC Med Res Methodol.

[17] Sandelowski M. Whatever happened to qualitative description? Res Nurs Health. 2000;23(4):334–340 [cited 2019 Feb 14]. Available from: http://doi.wiley.com/10.1002/1098-240X%28200008%2923%3A4%3C334%3A%3AAID-NUR9%3E3.0.CO%3B2-G. 10.1002/1098-240x(200008)23:4<334::aid-nur9>3.0.co;2-g 10940958

[18] Palys T, Given LM (2008). Purposive sampling. The Sage Encyclopedia of Qualitative Research Methods (Vol.2). In: Qualitative Research Methods.

[19] Saunders B, Sim J, Kingstone T, Baker S, Waterfield J, Bartlam B (2018). Saturation in qualitative research: exploring its conceptualization and operationalization. Qual Quant.

[20] Musselwhite K, Cuff L, McGregor L, King KM (2007). The telephone interview is an effective method of data collection in clinical nursing research: A discussion paper. Int J Nurs Stud.

[21] Alberta Queen’s Printer. Health Professions Act: PHARMACISTS AND PHARMACY TECHNICIANS PROFESSION REGULATION. Alberta; 2006. [cited 2019 May 26]. Available from: www.qp.alberta.ca.

[22] Attride-Stirling J (2001). Thematic networks: an analytic tool for qualitative research. Qual Res.

[23] Keene Woods N, Chesser AK (2017). Validation of a Single Question Health Literacy Screening Tool for Older Adults. Gerontol Geriatr Med.

[24] Quirk A, MacNeil V, Dhital R, Whittlesea C, Norman I, McCambridge J (2016). Qualitative process study of community pharmacist brief alcohol intervention effectiveness trial: Can research participation effects explain a null finding?. Drug Alcohol Depend.

[25] Donohue JM, Huskamp HA, Wilson IB, Weissman J (2009). Whom do older adults trust most to provide information about prescription drugs?. Am J Geriatr Pharmacother.

[26] Siddiqua A, Kareem Abdul W, Ayan S, Al Azm L, Ali S (2018). Antecedents of patients’ trust in pharmacists: empirical investigation in the United Arab Emirates. Int J Pharm Pract.

[27] Gregório J, Cavaco AM, Lapão LV (2017). How to best manage time interaction with patients? Community pharmacist workload and service provision analysis. Res Soc Adm Pharm.

[28] Sleath B (1995). Pharmacist Question-Asking in New Mexico Community. Am J Pharm Educ.

[29] Hamilton DW, De Salis I, Donovan JL, Birchall M (2013). The recruitment of patients to trials in head and neck cancer: a qualitative study of the EaStER trial of treatments for early laryngeal cancer. Eur Arch Oto-Rhino-Laryngology.

[30] De Salis I, Tomlin Z, Toerien M, Donovan J (2008). Using qualitative research methods to improve recruitment to randomized controlled trials: the quartet study. J Heal Serv Res Policy.

[31] Goel MS, Brown TL, Williams A, Cooper AJ, Hasnain-Wynia R, Baker DW (2011). Patient reported barriers to enrolling in a patient portal. J Am Med Informatics Assoc.

[32] Humphreys K, Maisel NC, Blodgett JC, Fuh IL, Finney JW (2013). Extent and reporting of patient nonenrollment in influencial randomized clinical trials, 2002 to 2010. JAMA Intern Med.

[33] Paramasivan S, Huddart R, Hall E, Lewis R, Birtle A, Donovan JL (2011). Key issues in recruitment to randomised controlled trials with very different interventions: a qualitative investigation of recruitment to the SPARE trial (CRUK/07/011). Trials.

[34] Chang B-H, Hendricks AM, Slawsky MT, Locastro JS (2004). Patient recruitment to a randomized clinical trial of behavioral therapy for chronic heart failure. BMC Med Res Methodol.

[35] Treweek S, Pitkethly M, Cook J, Fraser C, Mitchell E, Sullivan F, et al. Strategies to improve recruitment to randomised trials. Cochrane Datab Syst Rev. 2018;2018 John Wiley and Sons Ltd [cited 2020 Sep 15]. Available from: https://pubmed.ncbi.nlm.nih.gov/29468635/. 10.1002/14651858.MR000013.pub6 PMC7078793 29468635

[36] Canvin K, Jacoby A (2006). Duty, desire or indifference? A qualitative study of patient decisions about recruitment to an epilepsy treatment trial. Trials.

[37] Sheridan R, Martin-Kerry J, Hudson J, Parker A, Bower P, Knapp P (2020). Why do patients take part in research? An overview of systematic reviews of psychosocial barriers and facilitators. Trials.

[38] Campbell DJT, Manns BJ, Soril LJJ, Clement F. Comparison of Canadian public medication insurance plans and the impact on out-of-pocket costs. C Open [Internet]. 2017 Nov 22 [cited 2020 Sep 15];5(4):E808–13. Available from: www.cmajopen.ca/content/5/4/E808/. 10.9778/cmajo.20170065 PMC5741433 29180377

