# Supplementary material for: Pharmacist and patient perspectives on recruitment strategies for randomized controlled trials: a qualitative analysis
Source: BMC Med Res Methodol. 2020 Oct 31;20:270. doi: 10.1186/s12874-020-01140-6 (PMC7603682; doi:10.1186/s12874-020-01140-6)
Supplement: Supplementary file 1 — Additional file 1. Interview Guide - Assessing outcomes of enhanced Chronic disease Care through patient Education and a value-baSed formulary Study (ACCESS). [file 12874_2020_1140_MOESM1_ESM.docx]

Thank you for agreeing to participate in our interview today. We wish to discuss your experience working with participants from the ACCESS trial through your pharmacy. We will ask your experiences with the participants enrolled in our program, specifically experiences with MOXIE (our educational platform) and copayment elimination.

1. **Study knowledge**

Can you tell me what you know about the ACCESS trial?

- What do you think the study investigators trying to find out/what is the objective of the study?
- What are the interventions being used in the ACCESS study? (do they know anything about MOXIE?)
- How does a participant enroll in the study?
- What does participation entail? (do people have to travel to Calgary? Study follow-ups)
- How is one assigned to which intervention they are going to receive?
- What outcomes do you think the ACCESS study team is going to follow?

1. **Recruitment**

How did you hear about the ACCESS Study and what your initial interactions were like?

- How was the ACCESS study explained to you?
- Did you speak directly with a member of the ACCESS team? (Phone? Email? In person? Mail?)
- In your opinion how did the conversation go?
- Do you remember what your thoughts were initially?
- Was the study explained to you clearly?

Please describe the methods you used for recruiting your patients into the ACCESS study?

- Calling patients directly
- Speaking about the study to patients
- Posters in pharmacy
  - How often did you replace the posters
- Brochures on display in pharmacy
- Putting brochures in prescription bags
  - How did you decide which patients to target for brochures
- Full team at pharmacy aware of study?
- Do you think that there are any major barriers to recruitment?
- Have any patients mentioned the study to you?

In your opinion, how have patients responded to the methods of recruitment you have used in your pharmacy?

- How many patients do you think you attempted to recruit for the ACCESS Study?

1. **The Educational Intervention (MOXIE)**

What do you know about the educational intervention of the ACCESS Study?

- Have any of your patients ever spoken to you about MOXIE – or the cards they receive in the mail?
- How do patients feel about these? Have you heard any feedback about them?

Did any of your patients ever show you a letter in a blue envelope that they were asked to bring to their pharmacist?

- Did you read this letter? (With your patient? After they left?)
- What were your thoughts on the letter?
- How did you feel receiving a letter from your patients about their medications?
- Did any conversations about statins/ACE-ARBs start because of this letter?
- Did you ever fax the recommendations to the patient’s prescriber?
- Did any patients get started on one of these medications because of this letter?

Have any of your patients who are enrolled in the study spoken to you about blister packing/medication reviews since they joined?

- Did they mention whether this was recommended to them within the educational materials they received?
- Do you think this might be beneficial for their care? How?

Have any of your patients who are enrolled in the study who are smokers spoken to you about receiving support to help them quit smoking?

1. **Copayment Elimination Intervention (Blue Cross)**

Have any of your patients been randomized to receive free medication coverage? What are your thoughts on the impact this has had on the patients?

Have any of your patients had any trouble getting their coverage changed?

- Have any of the medications that should have been covered, not been covered?
- Did patients bring in the list of medications to be covered to review with you?
- Were any participants surprised/upset that not all their medications, or their testing supplies were not covered?

In your opinion, what are patients’ perceptions of receiving free medications?

Have any patients mentioned the impact that this has had for them?

Did you find this was a conversation starter with your patients about their medications?

How do patients respond when they were not randomized to receive free medication coverage?

Do you have any additional comments about the ACCESS trial/recruitment methods/interventions?

Thank you for participating in today’s interview. Using the information you provided, we will work on developing a qualitative research paper to describe experiences from the ACCESS study.
