# Supplementary material for: Pharmacist and patient perspectives on recruitment strategies for randomized controlled trials: a qualitative analysis
Source: BMC Med Res Methodol. 2020 Oct 31;20:270. doi: 10.1186/s12874-020-01140-6 (PMC7603682; doi:10.1186/s12874-020-01140-6)
Supplement: Supplementary file 2 — Additional file 2. Agenda - ACCESS Study-Recruitment material Focus Group. [file 12874_2020_1140_MOESM2_ESM.docx]

# AGENDA

| ACCESS Study-Recruitment material Focus Group | July 20, 2017 12pm – 4pm |
| --- | --- |

| Facilitators | Dave Campbell, Terry Saunders-Smith, Alexa Desjarlais, Gen Juillet (Cumming School of Medicine Marketing-Communications Advisor) |
| --- | --- |
| Attendees: | Access Study participants |
|  |  |
|  |  |

| 12PM – 1245PM | Lunch Registration  Consent  Introductions | TRWGE83 |
| --- | --- | --- |
| 1245PM – 1:15PM | Introductions/Ice Breaker Question: You are all receiving free medications. Has this helped you in the management of your chronic condition? If so, in what ways?  What has the savings meant to you?  What have you been able to do with the money that you otherwise would not have had?  Have you experienced any problems with your coverage since joining the study? | TRWGE83 |
| 1:15PM – 2:00PM | Impressions of current materials-Questions How did you hear about the study?  Where did you see our poster or brochure? *Show  What was you initial impression when you saw these materials?  What made you actually call the number provided?  Did you have reservations about calling?  How long did it take you from the time you heard about the study, until you actually called in to enroll? | TRWGE83 |
| 2:00PM – 2:15PM | Break | TRWGE83 |
| 2:15PM – 2:45PM | Open-ended Questions about how to improve We notice we are having a difficult time getting people to actually call us-why do you think this might be?  Is there something in our messaging that may scare people off?  What do you think should be the main take home point when people see the advertising?  What do you think would make someone like yourself call in and enroll in the study? | TRWGE83 |

| 2:45PM – 3:15PM | Participants provide ideas on paper We will give participants a piece of paper and ask them to create key messages that they believe will help with recruitment.  We will take all of the ideas and present one at a time and discuss | TRWGE83 |
| --- | --- | --- |
| 3:15PM – 4:00PM | Show new recruitment versions We will show brochure drafts to participants  We will show brochure messaging to the participants  We will ask each participant to rank both messaging and design from left to right in the order, how motivated you are to call in and participate  Open discussion about the different brochures  How well you relate to the pictures  Thoughts on the wording/main messaging/description  Color and Design | TRWGE83 |

If not answered:

What is more motivating to make people call in:

Helping others/and to better the system for all OR

Potential personal benefits ($ and Education).

# AGENDA

| ACCESS Study-Recruitment material Focus Group | August 3, 2017 1pm – 5pm |
| --- | --- |

| Facilitators | Dave Campbell, Terry Saunders-Smith, Sarah Gil |
| --- | --- |
| Attendees: | Access Study participants |
|  |  |
|  |  |

| 1PM – 1:45PM | Lunch Registration  Consent | TRWGE83 |
| --- | --- | --- |
| 1:45PM – 2:15PM | Introductions/Ice Breaker Overall how has your experience been with MOXIE so far?  What do you find to be the most helpful things about MOXIE?  What could be improved to make MOXIE more helpful in helping you become your better self?  Do you remember getting letters in your starter kit from Moxie? Did you take them to your Doctor/Pharmacist?  Why or why not? | TRWGE83 |
| 2:15PM – 3:00PM | Impressions of current materials-Questions How did you hear about the study?  Where did you see our poster or brochure? *Show  What was you initial impression when you saw these materials?  What made you actually call the number provided?  Did you have reservations about calling?  How long did it take you from the time you heard about the study, until you actually called in to enroll? | TRWGE83 |
| 3:00PM – 3:15PM | Break | TRWGE83 |
| 3:15PM – 3:45PM | Open-ended Questions about how to improve We notice we are having a difficult time getting people to actually call us-why do you think this might be?  Is there something in our messaging that may scare people off?  What do you think should be the main take home point when people see the advertising?  What do you think would make someone like yourself call in and enroll in the study? | TRWGE83 |

| 3:45PM – 4:15PM | Participants provide ideas on paper We will give participants a piece of paper and ask them to create key messages that they believe will help with recruitment.  We will take all of the ideas and present one at a time and discuss | TRWGE83 |
| --- | --- | --- |
| 4:15PM – 5:00PM | Show new recruitment versions We will shoe brochure drafts to participants  We will ask each participant to rank from left to right in the order, how motivated you are to call in and participate  Open discussion about the different brochures  How well you relate to the pictures  Thoughts on the wording/main messaging/description  Color and Design | TRWGE83 |

If not answered:

What is more motivating to make people call in:

Helping others/and to better the system for all OR

Potential personal benefits ($ and Education).
